# Discussions about reproductive and sexual health among young adult survivors of cancer

**DOI:** 10.1002/cam4.666

**Published:** 2016-02-21

**Authors:** Ying Wang, Leo Chen, Jenny Y. Ruan, Winson Y. Cheung

**Affiliations:** ^1^ British Columbia Cancer AgencyVancouverCanada

**Keywords:** Cancer, fertility preservation, reproductive health, sexual health, survivorship

## Abstract

Fertility preservation and sexual health are increasingly important as more young cancer patients survive their disease. Our aims were to describe the frequency with which reproductive and sexual health discussions occur, and to identify clinical factors associated with these discussions. Medical records of patients aged 20–39 diagnosed with solid tumors from 2008–2010 who survived ≥2 years were retrospectively reviewed. Multivariate logistic models were used to explore the relationship between clinical factors and occurrence of discussions. We analyzed 427 survivors: median age was 35 years, 29% were men, 88% had baseline [Eastern Cooperative Oncology Group (ECOG)] ECOG 0, and 79% were in a relationship. Only 58% and 7% of patients received discussions about reproductive and sexual health, respectively, at their initial oncology consultation, most of which were led by medical oncologists. There was a significant association between reproductive and sexual health conversations, in that those who engaged in dialog about one topic were more likely to participate in discussions about the other (*P* = 0.01). Patients with gynecologic malignancies (*P* < 0.0001) were more inclined to engage in sexual health discussions. Only a minority (19%) of patients took specific action toward fertility preservation, but the receipt of reproductive health discussions was a strong and independent driver for pursuing fertility preservation (*P* < 0.0001). The impact of cancer and its treatment on fertility and sexual health was inadequately addressed at the time of diagnosis among young cancer survivors. This warrants specific attention since having reproductive health discussions was strongly predictive of patients pursuing fertility preservation strategies.

## Introduction

It is estimated that one‐tenth of all new cancers are diagnosed in patients aged 20 to 39 years 1. With improvements in early detection and treatment, the majority of these individuals will overcome their disease to become cancer survivors 1. As this group of young adults transition from being patients to survivors, there is a growing need to address important issues pertaining to their long‐term follow‐up, such as their reproductive and sexual health. Incorporating discussions about these topics early in the course of their cancer is increasingly recognized as an important component of care even though long‐term repercussions of treatments may not always be seen as priorities when patients are first diagnosed with cancer and are still adjusting to the burden of a malignant diagnosis 2, 3. It is frequently only after treatment completion that patients face the reality of complications, such as infertility and sexual dysfunction 4, 5, 6, 7. Many of these patients experience regret and acknowledge in retrospect that they would have taken specific actions to better preserve their reproductive health, if they had been given the opportunity 6.

Treatments of specific tumors, such as genitourinary and gynecological malignancies can pose direct deleterious effects on fertility since therapies for these particular cancers can often involve chemical or physical removal of the reproductive organs 8. Many conventional chemotherapy agents can also temporarily or permanently harm reproductive health. In the setting of breast cancer, for instance, up to 80% of patients receiving chemotherapy may experience permanent amenorrhea and face the risk of premature ovarian failure 8, 9, 10. In addition, radiotherapy can have detrimental effects on gonadal function, depending on the dose and the field of irradiation 11, 12. One study showed a 20‐fold increased risk of ovarian failure in patients who received irradiation to their abdomen when compared to those who underwent irradiation to other areas 13. Cancer treatments can also lead to sexual dysfunction 14. Approximately, 30–100% of breast cancer survivors experience decreased sexual desire and arousal, vaginal dryness, anorgasmia, or dyspareunia 16, 17, 18, 19, 20, 21, 22, 23. In another recent study, 54% and 25% of male rectal and colon cancer survivors, respectively, reported erectile dysfunction 15.

Despite these significant consequences from cancer therapies, a study demonstrated that only 50% of young women with breast cancer recalled having a discussion on reproductive health risks with their healthcare providers prior to embarking on cancer treatments 24. An even lower proportion of male patients with colorectal cancer reported these discussions 25. Similarly, the frequency of sexual health discussions between physicians and cancer patients was also found to be low 26. Of note, prior studies have been small and focused primarily on survey studies which can be subject to recall bias. There has also been a lack of research on factors which predict the occurrence of fertility and sexual health discussions. Thus, the aims of the current population‐based study are to (1) determine the frequency at which reproductive and sexual health discussions occurred in common cancers, and (2) identify clinical factors associated with the occurrence of such conversations. Insights gained from this study can hopefully be used in the future to optimize the follow‐up care of young adult cancer survivors and to ensure that their fertility and sexual function concerns are adequately and promptly addressed.

## Methods

### Characteristics of the study setting

The British Columbia Cancer Agency is a provincial and population‐based cancer control program that is responsible for funding and providing cancer treatment to approximately 4.5 million residents in the province of British Columbia, Canada. At the time of this study, the agency was comprised of five regional cancer centers that were geographically distributed across different catchment areas of the province in order to ensure equitable access to cancer care irrespective of place of residence. All centers offer a full range of cancer programs including outpatient oncology clinics, chemotherapy suites, radiation facilities, surgical services, inpatient units, palliative and supportive care, and the opportunity to participate in clinical trials for the estimated 15,000–20,000 new patients referred to the British Columbia Cancer Agency annually. This study was conducted upon receiving full approval from the institutional Research Ethics Board.

### Description of the patient population

We conducted a retrospective chart review and included consecutive patients diagnosed with selected solid tumors from 2008 to 2010, who were aged 20–39 years at the time, referred to and evaluated at any of the British Columbia Cancer Agency regional cancer centers, and still alive at 2 or more years following their cancer diagnosis. We selected this specific age range as it encompassed a key demographic group in whom fertility may be particularly pertinent, but excluded patients younger than 20 years since these cases are still largely managed by pediatric oncologists in British Columbia. We defined survivors as those surviving beyond 2 years because most active anticancer treatments (except for hormonal therapies) are completed by this time point. This is also consistent with definitions used by prior investigators in existing published literature 27. We focused on solid tumors that have a growing and young survivorship population, specifically breast, testicular, and gynecological malignancies. Solid tumors that commonly affect older patients were excluded as these cases may have age‐related infertility and thus reproductive health discussions may be less applicable. Hematologic malignancies were further excluded since these are mainly treated at high‐volume transplant centers outside the British Columbia Cancer Agency.

### Measurement of outcomes and variables

The main outcome measures included whether a discussion on (1) reproductive health and (2) sexual health occurred at the first oncology consultation with a medical, radiation, or surgical oncologist, and if not, whether either of these topics was discussed within 6 months of the initial visit with any of the oncologists. All physicians at the British Columbia Cancer Agency are provided with standardized guidelines and formats for the dictation of medical notes. Charts were reviewed in full until the first documented conversation pertaining to reproductive and sexual health or until 6 months of the initial consultation, whichever came first. Study outcomes were obtained through evaluation of all electronic medical records by two authors (YW, JYR) who were blinded from each other to ensure consistency in interpretation of clinical information. Discordant interpretations between the two investigators were 3% and disagreements were resolved through consensus by involving the senior investigator (WYC) who conducted a separate review. For the purposes of this study, conversations about reproductive health were considered to have occurred if records described any of the following: current or future family planning, impact of treatment on fertility, or options regarding fertility preservation (e.g., sperm banking or egg retrieval). Likewise, sexual health discussions were deemed to have taken place if records described discussion of any of the following: treatment effects on sexual dysfunction, use of contraceptives, sexual activity during treatment, or interventions to manage symptoms of impotence or decreased libido. For the purposes of this study, fertility preservation was defined as (1) referral or involvement of fertility specialists or clinics to discuss options such as sperm banking in males or egg preservation in females, or (2) opting for fertility sparing treatments. Additional covariates were collected for analyses, such as patient demographics (e.g., gender, age at diagnosis), clinical characteristics (e.g., ECOG [Eastern Cooperative Oncology Group] performance status)^28^, and treatment parameters (e.g., modality of therapy, treatment location). We also considered relationship status, number of biological or adopted children, and cancer history in a first degree relative in the analysis because the presence of any one of these factors may increase patients' awareness of reproductive and sexual health issues and prompt them to proactively engage in conversations about these topics with their physicians.

### Statistical analyses

Patient demographics and clinical characteristics were summarized with descriptive statistics. The Chi‐squared and Fisher's exact tests were used to assess for variations in features between patient groups. Logistic regression models were constructed to examine for predictors of reproductive and sexual health discussions while adjusting for confounders. Covariates that were clinically significant as well as those found to be significantly associated with outcomes on univariate analyses were considered in a backwards selection procedure to refine the models such that covariates that did not significantly improve model fitness as per the likelihood ratio test were removed one at a time. Because results for discussions at baseline and at 6 months were similar, mainly findings at baseline are presented. All tests were two sided where a *P*‐value of <0.05 was considered statistically significant. The statistical software program SAS 9.3 (Cary, NC, USA) was used for all statistical analyses.

## Results

### Patient characteristics

We identified a total of 427 eligible patients. At the time of cancer diagnosis, the median age was 35 years (range 20–39), 124 (29%) were men, 375 (88%) had ECOG 0, 336 (79%) were in a relationship, 210 (49%) had children, and 122 (29%) reported a family history of cancer among first degree relatives. In this study cohort, patients were most commonly diagnosed with breast cancer (*N* = 225; 53%) followed by testicular cancer (*N* = 124; 29%), and gynecological malignancies (*N* = 78; 18%). Additional clinical characteristics are summarized in Table 1.

**Table 1:** Patient characteristics at time of initial consultation

Characteristics	*N*	%
Age		
<31	94	22.01
31–34	99	23.19
35–38	167	39.11
39+	67	15.69
Sex		
Female	303	70.96
Male	124	29.04
ECOG (Eastern Cooperative Oncology Group)		
0	375	87.82
1 or 2	52	12.18
Relationship Status		
Single	91	21.31
Partner	336	78.69
Children at initial consultation		
Had children	210	49.18
No children	217	50.82
Family history of cancer		
No	169	39.58
Yes	258	60.42
Family history of cancer among 1st degree relatives		
No	305	71.43
Yes	122	28.57
Comorbidities		
No	259	60.66
Yes	168	39.34
Tumor Group		
Breast	225	52.69
Testicular	124	29.04
Gynecological	78	18.27
Treatment Site		
Other	213	49.88
Teaching hospital	214	50.12
Chemotherapy planned		
No	147	34.43
Yes	280	65.57
Radiotherapy planned		
No	222	51.99
Yes	205	48.01
Surgery planned		
No	330	77.28
Yes	97	22.72

### Reproductive health discussions

In terms of patient–physician conversations, 249 (58%) had discussions about reproductive health at the initial consultation, with 66% of these held by medical oncologists. By 6 months, an additional 29 (7%) patients had undergone discussions about fertility of which most (93%) were also led by medical oncologists (Table 2). On univariate analyses, patients already in relationships and those with a known family history of cancer among first degree relatives were more likely to have had discussions about their fertility at the initial consultation (*P* = 0.03 and *P* = 0.05, respectively). In both univariate and multivariate analyses, there was also a significant association between reproductive health conversations and sexual health conversations in that those who engaged in dialog about one topic were more likely to participate in discussions about the second topic (univariate: *P* = 0.01, multivariate: OR 3.06, 95% CI 1.20–7.87, *P* = 0.02). Tables 3 and 4 highlight in detail the clinical factors associated with the occurrence of a reproductive health discussion on univariate and multivariate analyses, respectively.

**Table 2:** Frequency of sexual and reproductive health discussions conducted by medical (MO), radiation (RO), and surgical oncologists (SO)

Characteristics	Frequency of discussions	% of Patients with discussions	Distribution of discussions by MO versus. RO/SO
Sexual health at initial consultation	32/427	7.49%	41% versus 59%
Sexual Health within 6 months after initial consultation	50/427	11.71%	67% versus 33%^a^
Reproductive health discussion at initial consultation	249/427	58.31%	66% versus 34%
Reproductive health discussions within 6 months after initial consultation	278/427	65.11%	93% versus 7%^b^

a Percentage distribution reflects the additional 18 patients who had a sexual health discussion by 6 months.

b Percentage distribution reflects the additional 29 patients who had a reproductive health discussion by 6 months.

**Table 3:** Univariate analysis of factors associated with fertility and sexual health discussions, and fertility preservation

			Fertility Discussion			Sexual Health Discussion			Fertility Preservation		
Category	Level	Total *N*	*N*	%	*P*‐Value	*N*	%	*P*‐Value	*N*	%	*P*‐Value
Age+	<31	94	52	55.3	0.69	3	3.2	0.02	29	30.9%	**<0.0001**
	31–34	99	60	60.6		10	10.1		28	28.3%	
	35–38	167	101	60.5		9	5.4		23	13.8%	
	39+	67	36	53.7		10	14.9		4	6.0%	
Sex	Female	303	182	60.1	0.25	26	8.6	0.18	45	14.9%	**<0.0001**
	Male	124	67	54.0		6	4.8		39	31.5%	
ECOG+	0	375	222	59.2	0.32	28	7.5	0.95	78	20.8%	0.12
	1 or 2	52	27	51.9		4	7.7		6	11.5%	
Relationship Status	Single	91	44	48.4	**0.03**	7	7.7	0.94	24	26.4%	0.07
	Partner	336	205	61.0		25	7.4		60	17.9%	
Children at Initial Consultation	Had children	210	119	56.7	0.50	16	7.62	0.92	19	9.05%	**<0.0001**
	No children	217	130	59.9		16	7.37		65	30.0%	
Family history of cancer	No	169	91	53.9	0.13	12	7.1	0.80	28	16.6%	0.19
	Yes	258	158	61.2		20	7.8		56	21.7%	
Family history of cancer Among 1st degree relatives+	No	305	169	55.4	**0.05**	17	5.6	**0.02**	62	20.3%	0.59
	Yes	122	80	65.6		15	12.3		22	18.0%	
Comorbidities	No	259	151	58.3	0.99	17	6.6	0.36	57	22.0%	0.13
	Yes	168	98	58.3		15	8.9		27	16.1	
Tumor Group+	Breast	225	129	57.3	0.14	11	4.9	**<0.0001**	24	10.7	**<0.0001**
	Testicular	124	67	54.0		6	4.8		39	31.5	
	Gynecological	78	53	67.9		15	19.2		21	26.9	
Treatment Site	Other	213	126	59.2	0.73	14	6.6	0.47	32	15.0%	**0.02**
	Teaching hospital	214	123	57.5		18	8.4		52	24.3	
Chemotherapy Planned	No	147	91	61.9	0.38	12	8.2	0.70	33	22.4	0.30
	Yes	280	159	56.8		20	7.1		51	18.2	
Radiotherapy planned	No	222	134	60.4	0.37	12	5.4	0.09	62	27.9	**<0.0001**
	Yes	205	115	56.1		20	9.8		22	10.7	
Surgery planned	No	330	187	56.7	0.20	24	7.3	0.75	58	17.6	**0.04**
	Yes	97	62	63.9		8	8.2		26	26.8	
Sexual health discussions at initial consultation	No	395	223	56.5	**0.01**	**–**	**–**	**–**	**–**	**–**	**–**
	Yes	32	26	81.3		**–**	**–**		**–**	**–**	

Bold values represent statistically significant values on univariate analysis.

**Table 4:** Multivariate analysis of factors associated with fertility and sexual health discussions, and fertility preservation

		Fertility discussion				Sexual health discussion				Fertility preservation			
Category	Level	OR	95% CI	*P*‐Value	Global *P* ‐Value	OR	95% CI	*P* ‐Value	Global *P*‐Value	OR	95% CI	*P*‐Value	Global *P*‐Value
Age+	<31	1	–	–	–	1	–	–	–	1	–	–	–
	31–34	1.05	0.57–1.93	0.61	0.68	3.72	0.94–14.72	0.25	0.05	0.69	0.31–1.49	0.16	**0.01**
	35–38	1.06	0.60–1.89	0.50		2.01	0.51–7.99	0.48		0.43	0.20–0.91	0.73	
	39+	0.74	0.37–1.47	0.24		5.58	1.37–22.76	0.02		0.16	0.05–0.55	0.01	
ECOG+	0	1	–	–	–	1	–	–	–	1	–	–	–
	1 or 2	1.27	0.70–2.33	0.43	–	1.03	0.33–3.24	0.96	–	1.83	0.65–5.17	0.25	–
Relationship status	Single	1	–	–	–	–	–	–	–	–	–	–	–
	Partner	1.59	0.97–2.60	0.06	–	–	–	–	–	–	–	–	–
Children at initial consultation	Had children	–	–	–	–	–	–	–	–	1	–	–	–
	No children	–	–	–	–	–	–	–	–	3.13	1.64–5.98	**0.0005**	–
Family History of cancer among 1st degree relatives+	No	1	–	–	–	1	–	–	–	–	–	–	–
	Yes	1.37	0.87–2.16	0.17	–	1.82	0.84–3.98	0.13	–	–	–	–	–
Tumor group+	Breast	1	–	–	–	1	–	–	–	1	–	–	–
	testicular	0.94	0.58–1.53	0.31	0.35	1.38	0.47–4.03	0.32	**0.001**	3.43	1.74–6.78	0.007	**0.002**
	Gynecological	1.44	0.81–2.57	0.15		5.13	2.17–12.12	0.001		2.11	0.97–4.59	0.72	
Treatment site	Other	–	–	–	–	–	–	–	–	1	–	–	–
	Teaching hospital	–	–	–	–	–	–	–	–	1.80	0.97–3.56	0.06	–
Sexual health discussion at initial consultation	No	1	–	–	–	–	–	–	–	–	–	–	–
	Yes	3.06	1.20–7.87	0.02	–	–	–	–	–	–	–	–	–
Fertility discussion at Initial consultation	No	–	–	–	–	–	–	–	–	1	–	–	–
	Yes	–	–	–	–	–	–	–	–	13.06	5.75–29.62	**<0.0001**	–

Note that a backwards selection procedure was used to refine the models so that covariates that did not significantly improve model fitness as per the likelihood ratio test were removed one at a time.

ECOG, Eastern Cooperative Oncology Group.

Bold values represent statistically significant values on multivariate analysis.

### Sexual health discussions

Only 32 (7%) patients had a conversation about sexual health at their initial consultation, with 59% of these initiated by radiation or surgical oncologists. An additional 18 (4%) patients received a sexual health discussion by 6 months of which the majority (67%) were conducted by medical oncologists (Table 2).

On univariate analysis, those with a known family history of cancer among first‐degree relatives were more likely to have had sexual health conversations (*P* = 0.02). In addition, gynecological patients engaged in sexual health discussions more frequently than breast or testicular patients (*P* < 0.0001). In multivariate analysis, individuals affected by gynecological malignancies continued to experience significantly higher odds of having a sexual health discussion when compared to their counterparts (OR 5.13, 95% CI 2.17–12.12, *P* = 0.001). Factors associated with sexual health discussions on univariate and multivariate analyses are summarized in Table 3 and Table 4, respectively.

### Taking action toward fertility preservation

In the entire cohort, 84 (19%) patients took action toward fertility preservation of whom 17 (20%) opted for fertility sparing treatments and 32 (38%) were further referred to reproductive specialists. Of the 17 patients who opted for fertility sparing treatments, 12 cervical cancer patients underwent vaginal trachelectomy instead of standard hysterectomy, four endometrial cancer patients received high‐dose progesterone therapy rather than surgery, and one ovarian cancer patient kept her contralateral ovary in order to preserve future fertility. Tables 3 and 4 show clinical factors associated with fertility preservation. On univariate analysis, older individuals (*P* < 0.0001), females (*P* < 0.0001), and patients already with children (*P* < 0.0001) were less likely to take specific actions to protect their fertility. Although patients who were undergoing surgery were more likely to undergo fertility preservation than those in whom surgery was not planned (*P* = 0.04), patients who were receiving radiation were actually less likely to pursue fertility preservation than those not being treated with radiation (*P* < 0.0001). In addition, patients affected by testicular and gynecological cancers were more inclined to undergo fertility preservation than patients with breast cancer (*P* < 0.0001), as were those treated at teaching hospitals versus nonteaching hospitals (*P* = 0.02).

On multivariate analysis, similar findings were observed in that patients in the oldest age group were less likely to pursue fertility preservation (OR 0.16, 95% CI 0.05–0.55, *P* = 0.01). Conversely, individuals with testicular and gynecological cancers (OR 3.43, 95% CI 1.74–6.78 and OR 2.11, 95% CI 0.97–4.59, respectively, *P* = 0.002), patients with no children, and those with a prior reproductive health discussion (OR 3.13, 95% CI 1.64–5.98, *P* = 0.0005 and OR 13.06, 95% CI 5.75–29.62, *P* < 0.0001, respectively) had significantly higher odds of proceeding with actions to preserve their fertility. There was also a persistent trend for patients seen at teaching hospitals to pursue fertility preservation more than those seen at nonteaching centers (OR 1.80, 95% CI 0.97–3.56, *P* = 0.06). Having a reproductive health discussion was strongly correlated with fertility preservation (Tables 3 and 4).

## Discussion

This study represents one of the largest analyses to describe the frequency of fertility and sexual health discussions in young adult cancer survivors of common solid tumors. Contrary to the recommendations from the American Society of Clinical Oncology (ASCO) 8 which highlight the importance of addressing reproductive and sexual health needs, our results indicate that discussions of these issues occur inadequately. This pattern was observed at the first consultation prior to initiation of cancer treatment, and persisted after six months. Specific factors such as relationship status and tumor site were correlated with engaging in these conversations. In turn, conversations about reproductive health were significantly associated with an increased likelihood of pursuing options for fertility preservation.

Prior research suggests that there may be a discrepancy between patients' and oncologists' perceptions of fertility discussions. Although surveys of patients indicate that only 34–72% report participating in reproductive health conversations 3, 6, 10, 24, 29, 30, similar surveys of oncologists report that 95–97% of physicians routinely discussed the potential impact of cancer treatment on fertility 31, 32. This apparent disconnect may be due in part to the recall bias inherent to surveys 3, potential barriers in patient–physician communication 33, or different views between patients and providers about the aspects of fertility and sexual function that actually warrant dialog 34, 35. Importantly, our study confirms that the frequency of clinically documented discussions about reproductive and sexual health is more consistent with the patient's recall than the physician's, underscoring that there are significant unmet needs 8, 29, 33.

Among those who had a reproductive health discussion, one noteworthy finding is that most occurred during the initial consultation before cancer treatment started. To some extent, this is reassuring because early conversations allow an opportunity for patients to fully consider options for fertility preservation 8. As shown in this as well as other studies 36, 37, 38, a significant proportion of patients who participated in reproductive health discussions subsequently pursued options to preserve their fertility. After adjusting for confounders, having a discussion was the strongest predictor of fertility preservation. This underscores the value of these conversations and the missed opportunity that may occur if adequate dialog fails to take place before cancer treatment is initiated. Many patients who are not given this opportunity experience regret 39.

Conversations about sexual health were particularly deficient even though research indicates that this is a significant concern among patients 3. Reasons for the lack of discussion may include poor awareness of treatment options for sexual function 40, time and resource limitations to address the topic appropriately 35, and potential concerns regarding the discomfort and negative rapport that may result if such conversations are not conducted sensitively 31, 41, 42. In this study, there was a correlation between reproductive and sexual health conversations in that those who engaged in dialog about one topic were more likely to participate in discussions about the other, suggesting that comfort and openness about these delicate issues can have a significant impact on willingness to discuss them 8, 43, and should be a focus of future quality improvement.

We observed a relationship between tumor site and fertility preservation where patients with testicular or gynecological cancers were more inclined to proceed with strategies to protect their fertility. Because management for these tumor sites can frequently involve removal of or modification to the reproductive anatomy, this may serve as strong motivation for patients and physicians to more openly discuss fertility implications. The perceived ease and lower costs of sperm banking when compared to egg retrieval and cryopreservation may further explain why testicular cancer patients were most likely to embark on fertility preservation 44. With advances in fertility preservation, however, the number of available options is increasing while the costs are decreasing, and thus should encourage discussions across all tumor groups.

Additional factors that were associated with discussions and pursuit of fertility preservation included individuals in a relationship and patients treated at teaching hospitals, respectively. These observations highlight that there could be potential biases in the way patients and physicians approach fertility. While those with a partner or spouse may be viewed conventionally as having more desire or readiness to discuss reproductive health 45, an increasing number of individuals opt for single parenthood at a later stage in their lives because of specific personal, lifestyle, and professional reasons 46. Further, fertility preservation remains a highly specialized therapeutic area and a stronger propensity to pursue fertility preservation options at teaching hospitals suggests that access to fertility services may be an ongoing barrier. These disparities indicate a need to develop appropriate models of care delivery that can standardize the way in which fertility is addressed across all demographic and geographic groups.

Interestingly, our results did not reflect any statistically significant sex difference in the likelihood of having reproductive and sexual health discussions. This is in contrast to a survey study by Armuand et al., which reported significantly greater proportion of males having had such discussions when compared to females 47. A possible explanation for this discrepancy could be the difference in the study cohorts. While our study reviewed patients with breast, testicular, or gynecological malignancies, Armuand et al. also included those with lymphoma and leukemia in the analysis 47. Moreover, the survey study was conducted in Sweden 47, which possibly had a different set of factors influencing the likelihood of fertility discussions. In addition, recall bias which is common in surveys was likely present in the study of Armuand et al., since they also uncovered inconsistencies between physician and patient report of fertility discussions 47.

Our findings should be interpreted in the context of several limitations. First, it is possible that the true frequency of reproductive or sexual health discussions was underestimated since conversations may have occurred without them being documented. However, it is reasonable to expect that any discussions of significant length or detail would appear in the medical records as would any other pertinent topics that were reviewed with the patient by the physician. Clinical documentation of discussions at the time they occurred is more accurate and less variable than physician and patient recall, respectively 48, 49. Second, this study was limited to the evaluation of three prevalent tumor types, namely breast, testicular, and gynecological cancers, so our findings may not be generalizable to other malignancies that can occasionally affect young adults, such as lymphoma or melanoma. Likewise, we limited our study cohort to those aged 20–39 years, so our results may not be applicable to older individuals in whom reproductive and sexual health may be equally important. Further, since all patients who opted for fertility sparing treatments were those faced with surgical removal of their reproductive organs, we were not able to provide insights on the impact of chemotherapy and radiation on patient treatment preferences. However, these limitations must also be weighed against the study's strengths, which include its relatively large sample size as well as its examination of other young adult malignancies in addition to breast cancer.

In conclusion, the impact of cancer and its treatment on fertility and sexual function was inadequately addressed with patients at the time of their diagnosis, even though such discussions appear to provide patients with increased opportunities to pursue fertility preservation strategies. There were disparities in reproductive and sexual health discussions within specific demographic groups, tumor sites, and treatment locations even though these issues are important for most patients. Better integration of these topics into our routine interactions with patients should be a priority and can mitigate the perceived discomfort surrounding reproductive and sexual health. This is particularly relevant in light of the increasing number of effective treatment options for infertility and sexual dysfunction that are available for the growing number of cancer survivors.

## Conflicts of Interest

The authors of this manuscript declare no conflicts of interest.

## References

[1] Canadian Cancer Society's Advisory Committee on Cancer Statistics . 2013 Canadian cancer statistics. Canadian Cancer Statistics 2013, Toronto, ON.

[2] Peate, M. , B. Meiser , M. Friedlander , et al. 2011 It's now or never: fertility‐related knowledge, decision‐making preferences, and treatment intentions in young women with breast cancer–an Australian fertility decision aid collaborative group study. J. Clin. Oncol. 29:1670–1677. 21444865 10.1200/JCO.2010.31.2462

[3] Ruddy, K. J. , S. I. Gelber , R. M. Tamimi , et al. 2014 Prospective study of fertility concerns and preservation strategies in young women with breast cancer. J. Clin. Oncol. 32:1151–1156. 24567428 10.1200/JCO.2013.52.8877 PMC4164759

[4] Blumenfeld, Z. 2012 Chemotherapy and fertility. Best Pract. Res. Clin. Obstet. Gynaecol. 26:379–390. 22281514 10.1016/j.bpobgyn.2011.11.008

[5] Fleischer, R. T. , B. J. Vollenhoven , and G. C. Weston . 2011 The effects of chemotherapy and radiotherapy on fertility in premenopausal women. Obstet. Gynecol. Surv. 66:248–254. 21756407 10.1097/OGX.0b013e318224e97b

[6] Partridge, A. H. , S. Gelber , J. Peppercorn , et al. 2004 Web‐based survey of fertility issues in young women with breast cancer. J. Clin. Oncol. 22:4174–4183. 15483028 10.1200/JCO.2004.01.159

[7] Carter, J. , K. Rowland , D. Chi , et al. 2005 Gynecologic cancer treatment and the impact of cancer‐related infertility. Gynecol. Oncol. 97:90–95. 15790443 10.1016/j.ygyno.2004.12.019

[8] Lee, S. J. , L. R. Schover , A. H. Partridge , et al. 2006 American Society of Clinical Oncology recommendations on fertility preservation in cancer patients. J. Clin. Oncol. 24:2917–2931. 16651642 10.1200/JCO.2006.06.5888

[9] Partridge, A. H. , H. J. Burstein , and E. P. Winer . 2001 Side effects of chemotherapy and combined chemohormonal therapy in women with early‐stage breast cancer. J. Natl. Cancer Inst. Monogr. 30:135–142. 11773307 10.1093/oxfordjournals.jncimonographs.a003451

[10] Duffy, C. M. , S. M. Allen , and M. A. Clark . 2005 Discussions regarding reproductive health for young women with breast cancer undergoing chemotherapy. J. Clin. Oncol. 23:766–773. 15681520 10.1200/JCO.2005.01.134

[11] Meirow, D. , and D. Nugent . 2001 The effects of radiotherapy and chemotherapy on female reproduction. Hum. Reprod. Update. 7:535–543. 11727861 10.1093/humupd/7.6.535

[12] Wasilewski‐Masker, K. , K. D. Seidel , W. Leisenring , et al. 2014 Male infertility in long‐term survivors of pediatric cancer: a report from the childhood cancer survivor study. J. Cancer Surviv. 2014 Sep;8(3):437–447. 24711092 10.1007/s11764-014-0354-6 PMC4276596

[13] Stillman, R. J. , J. S. Schinfeld , I. Schiff , et al. 1981 Ovarian failure in long‐term survivors of childhood malignancy. Am. J. Obstet. Gynecol. 139:62–66. 7457523 10.1016/0002-9378(81)90413-0

[14] Averyt, J. C. , and P. W. Nishimoto . 2014 Addressing sexual dysfunction in colorectal cancer survivorship care. J. Gastrointest. Oncol. 5:388–394. 25276411 10.3978/j.issn.2078-6891.2014.059 PMC4173048

[15] Den Oudsten, B. L. , M. K. Traa , M. S. Thong , et al. 2012 Higher prevalence of sexual dysfunction in colon and rectal cancer survivors compared with the normative population: a population‐base study. Eur. J. Cancer 48:3161–3170. 22608772 10.1016/j.ejca.2012.04.004

[16] Sadovsky, R. , R. Basson , M. Krychman , et al. 2010 Cancer and sexual problems. J. Sex Med. 7(1 Pt 2):349–373. 20092444 10.1111/j.1743-6109.2009.01620.x

[17] Fobair, P. , S. L. Stewart , S. Chang , C. D'Onofrio , P. J. Banks , and J. R. Bloom . 2006 Body image and sexual problems in young women with breast cancer. Psychooncology 15:579–594. 16287197 10.1002/pon.991

[18] Kedde, H. , H. B. van de Wiel , W. C. Weijmar Schultz , and C. Wijsen . 2013 Sexual dysfunction in young women with breast cancer. Support. Care Cancer 21:271–280. 22714701 10.1007/s00520-012-1521-9

[19] Bloom, J. R. , S. L. Stewart , S. Chang , and P. J. Banks . 2004 Then and now: quality of life of young breast cancer survivors. Psychooncology 13:147–160. 15022150 10.1002/pon.794

[20] Krychman, M. L. , L. Pereira , J. Carter , and A. Amsterdam . 2006 Sexual oncology: sexual health issues in women with cancer. Oncology 71:18–25. 17347586 10.1159/000100521

[21] Broeckel, J. A. , C. L. Thors , P. B. Jacobsen , M. Small , and C. E. Cox . 2002 Sexual functioning in long‐term breast cancer survivors treated with adjuvant chemotherapy. Breast Cancer Res. Treat. 75:241–248. 12353813 10.1023/a:1019953027596

[22] Bredart, A. , S. Dolbeault , A. Savignoni , et al. 2011 Prevalence and associated factors of sexual problems after early‐stage breast cancer treatment: results of a French exploratory survey. Psychooncology 20:841–850. 20568085 10.1002/pon.1789

[23] Dorval, M. , E. Maunsell , L. Deschenes , J. Brisson , and B. Masse . 1998 Long‐term quality of life after breast cancer: comparison of 8‐year survivors with population controls. J. Clin. Oncol. 16:487–494. 9469332 10.1200/JCO.1998.16.2.487

[24] Niemasik, E. E. , J. Letourneau , D. Dohan , et al. 2012 Patient perceptions of reproductive health counseling at the time of cancer diagnosis: a qualitative study of female California cancer survivors. J. Cancer Surviv. 6:324–332. 22752834 10.1007/s11764-012-0227-9

[25] Kumar, A. , A. Merali , G. R. Pond , and K. Zbuk . 2012 Fertility risk discussions in young patients diagnosed with colorectal cancer. Curr. Oncol. 19:155–159. 22670094 10.3747/co.19.942 PMC3364765

[26] Sporn, N. J. , K. B. Smith , W. F. Pirl , I. T. Lennes , K. A. Hyland , and E. R. Park . 2014 Sexual health communication between cancer survivors and providers: how frequently does it occur and which providers are preferred? Psychoncology 24:1167–1173. 10.1002/pon.3736 25534170

[27] Oken, M. , R. Creech , D. Tormey , et al. 1982 Toxicity and response criteria of the Eastern Cooperative Oncology Group. Am. J. Clin. Oncol. 5:649–655. 7165009

[28] Bouhnik, A. D. , M. K. Benediane , S. Cortaredona , et al. 2015 The labour market, psychosocial outcomes and health conditions in cancer survivors: protocol for a nationwide longitudinal survey 2 and 5 years after cancer diagnosis (the VICAN survey). Br. Med. J. Open 5:e005971. 10.1136/bmjopen-2014-005971 PMC4386221 25805526

[29] Thewes, B. , B. Meiser , A. Taylor , et al. 2005 Fertility‐ and menopause‐related information needs of younger women with a diagnosis of early breast cancer. J. Clin. Oncol. 23:5155–5165. 16051957 10.1200/JCO.2005.07.773

[30] Armuand, G. M. , K. A. Rodriguez‐Wallberg , L. Wettergren , et al. 2012 Sex differences in fertility‐related information received by young adult cancer survivors. J. Clin. Oncol. 20:2147–2153. 22585695 10.1200/JCO.2011.40.6470

[31] Adams, E. , E. Hill , and E. Watson . 2013 Fertility preservation in cancer survivors: a national survey of oncologists' current knowledge, practice and attitudes. Br. J. Cancer 108:1602–1615. 23579214 10.1038/bjc.2013.139 PMC3668471

[32] Forman, E. J. , C. K. Anders , and M. A. Behera . 2010 A nationwide survey of oncologists regarding treatment‐related infertility and fertility preservation in female cancer patients. Fertil. Steril. 94:1652–1656. 19945099 10.1016/j.fertnstert.2009.10.008

[33] Quinn, G. P. , S. T. Vadaparampil , B. A. Bell‐Ellison , B. A. Bell‐Ellison , C. K. Gwede , and T. L. Albrecht . 2008 Patient‐physician communication barriers regarding fertility preservation among newly diagnosed cancer patients. Soc. Sci. Med. 66:784–789. 18023955 10.1016/j.socscimed.2007.09.013

[34] Quinn, G. , S. T. Vadaparampil , P. Jacobsen , et al. 2009 Physician referral for fertility preservation in oncology patients: a national study of practice behaviors. J. Clin. Oncol. 27:5952–5957. 19826115 10.1200/JCO.2009.23.0250

[35] Quinn, G. P. , S. T. Vadaparampil , C. K. Gwede , et al. 2007 Discussion of fertility preservation with newly diagnosed patients: oncologists' views. J. Cancer Surviv. 1:146–155. 18648955 10.1007/s11764-007-0019-9

[36] Letouneau, J. M. , E. E. Ebbel , P. P. Katz , et al. 2012 Pre‐treatment fertility counseling and fertility preservation improve quality of life in reproductive age women with cancer. Cancer 118:1717. 10.1002/cncr.26459 PMC3235264 21887678

[37] Bastings, L. , O. Baysal , C. C. Beerendonk , D. D. Braat , and W. L. Nelen . 2014 Referral for fertility preservation counselling in female cancer patients. Hum. Reprod. 29:2228–2237. 25069500 10.1093/humrep/deu186

[38] Schover, L. R. , K. Brey , A. Lichtin , L. I. Lipshultz , and S. Jeha . 2002 Knowledge and experience regarding cancer, infertility, and sperm banking in younger male survivors. J. Clin. Oncol. 20:1880–1889. 11919248 10.1200/JCO.2002.07.175

[39] Thewes, B. , B. Meiser , J. Rickard , and M. Friedlander . 2003 The fertility‐ and menopause‐related information needs of younger women with a diagnosis of breast cancer: a qualitative study. Psychooncology 12:500–511. 12833562 10.1002/pon.685

[40] Peate, M. , B. Meiser , M. Friedlander , et al. 2001 It's now or never: fertility‐related knowledge, decision‐making preferences, and treatment intentions in young women with breast cancer—an Australian fertility decision aid collaborative group study. J. Clin. Oncol. 29:1670–1677. 21444865 10.1200/JCO.2010.31.2462

[41] Shimizu, C. , H. Bando , T. Kato , Y. Mizota , S. Yamamoto , and Y. Fujiwara . 2013 Physicians' knowledge, attitude, and behavior regarding fertility issues for young breast cancer patients: a national survey for breast care specialists. Breast Cancer. 20:230–240. 22271066 10.1007/s12282-011-0328-8

[42] Shien, T. , M. Nakatsuka , and H. Doihara . 2014 Fertility preservation in breast cancer patients. Breast Cancer 21:651–655. 23526260 10.1007/s12282-013-0463-5 PMC4210658

[43] Quinn, G. P. , S. T. Vadaparampil , L. King , et al. 2009 Impact of physicians' personal discomfort and patient prognosis on discussion of fertility preservation with young cancer patients. Patient Educ. Couns. 77:338–343. 19796912 10.1016/j.pec.2009.09.007

[44] Gunasheela, D. , and S. Gunasheela . 2014 Strategies for fertility preservation in young patients with cancer: a comprehensive approach. Indian J. Surg. Oncol. 5:17–29. 24669162 10.1007/s13193-014-0291-x PMC3964233

[45] Murphy, D. , E. Orgel , A. Termuhlen , S. Shannon , K. Warren , and G. P. Quinn . 2013 Why healthcare providers should focus on the fertility of AYA cancer survivors: it's not too late!. Front Oncol. 3:248. 24109589 10.3389/fonc.2013.00248 PMC3791875

[46] Baker, D. G. 1999 The increase of single parent families: an examination of causes. Policy Sci. 32:175–188.

[47] Armuand, G. M. , K. A. Rodriguez‐Wallberg , L. Wettergren , et al. 2012 Sex differences in fertility‐related information received by young adult cancer survivors. J. Clin. Oncol. 30:2147–2153. 22585695 10.1200/JCO.2011.40.6470

[48] Kerr, Z. Y. , J. P. Mihalik , K. M. Guskiewicz , W. D. Rosamond , K. R. Evenson , and S. W. Marshall . 2015 Agreement between athlete‐recalled and clinically documented concussion histories in former collegiate athletes. Am. J. Sports Med. 43:606–612. 25560539 10.1177/0363546514562180

[49] Litwin, M. S. , and K. A. Mcguigan . 1999 Accuracy of recall in health‐related quality‐of‐life assessment among men treated for prostate cancer. J. Clin. Oncol. 17: (abstr 2882)28882–28888. 10.1200/JCO.1999.17.9.2882 10561366

